# Occurrence and genetic characterization of *Toxoplasma gondii* and *Neospora caninum* in slaughtered domestic rabbits in central China

**DOI:** 10.1051/parasite/2019035

**Published:** 2019-06-14

**Authors:** Weifeng Qian, Wenchao Yan, Chaochao Lv, Rongzhen Bai, Tianqi Wang

**Affiliations:** 1 College of Animal Science and Technology, Henan University of Science and Technology No. 263 Kaiyuan Road, Luolong District Luoyang 471003 PR China

**Keywords:** *Toxoplasma gondii*, *Neospora caninum*, Rabbit, Genetic characterization

## Abstract

Currently, information on the occurrence and genetic characterization of *Toxoplasma gondii* and *Neospora caninum* in tissues of rabbits in China is lacking. In this study, brain and heart samples from 470 slaughtered domestic rabbits were collected in Henan Province, Central China. The occurrence rate of *T. gondii* and *N. caninum* DNA detected by nested PCR was 2.8% and 2.1%, respectively. There were no significant differences (*p* > 0.05) in the frequency of the two parasite infections in relation to sex, breed, and region. Three out of 13 *T. gondii*-positive samples were completely or partially genotyped at 11 genetic markers using PCR-RFLP, and one was identified as ToxoDB genotype #9. For *N. caninum*, three different sequences at the ITS1 region and two genotypes at the MS5 microsatellite locus were identified. To our knowledge, this is the first genetic characterization of *N. caninum* isolates from rabbits.

## Abstract (fr)

Nous manquons actuellement d’informations sur la présence et la caractérisation génétique de *Toxoplasma gondii* et *Neospora caninum* dans les tissus de lapins en Chine. Dans cette étude, des échantillons de cerveau et de cœur de 470 lapins domestiques abattus ont été recueillis dans la province du Henan, dans le centre de la Chine. Le taux de présence d’ADN de *T. gondii* et *N. caninum*, détecté par PCR imbriquée, était de 2,8 % et 2,1 %, respectivement. Il n’y avait pas de différence significative (*p* > 0,05) dans la fréquence des deux infections parasitaires en fonction du sexe, de la race et de la région du lapin. Trois des 13 échantillons positifs pour *T. gondii* ont été complètement ou partiellement génotypés au moyen de PCR-RFLP sur 11 marqueurs génétiques, et un a été identifié comme étant le génotype #9 de ToxoDB. Pour *N. caninum*, trois séquences différentes de la région ITS1 et deux génotypes du locus microsatellite MS5 ont été identifiés. À notre connaissance, il s’agit de la première caractérisation génétique d’isolats de *N. caninum* provenant de lapins.

## Introduction

*Toxoplasma gondii* infections are widely prevalent in warm-blooded vertebrates, including humans and rabbits worldwide, and can cause life-threatening toxoplasmosis in immunocompromized individuals. Fatal cases of toxoplasmosis in domestic rabbits have been reported in a few countries [6, 8]. It is estimated that up to one-third of the world’s human population has been infected with *T. gondii* [8]. The consumption of undercooked or raw meat containing tissue cysts is the primary risk factor for human *T. gondii* infections [8, 25]. Rabbit meat is one of the most nutritional white meats, and is very popular in China. Rabbits can be infected by ingestion of food or water contaminated with *T. gondii* oocysts from feline excrement, or by transplacental transmission of *T. gondii* to offspring [8]. Humans may become infected by eating undercooked rabbit meat, or from hand-to-mouth processes after slaughtering, skinning rabbits or dealing with undercooked or raw rabbit meat [1]. So far, only a few surveys have focused on *T. gondii* infection in domestic and wild rabbits in China [4, 13, 15, 24, 27]. However, information on the prevalence of *T. gondii* DNA in the tissues of domestic rabbits in China was not available.

*Neospora caninum* is similar to *T. gondii* in morphology and life cycle, and is one of the most important causes of abortion in cattle worldwide [7]. Canids are the definitive hosts, whereas many other animal species, including rabbits, are intermediate hosts [9]. There are no natural or experimental data on neosporosis in rabbits. To date, there has been only one report on the seroprevalence and DNA detection of *N. caninum* in wild rabbits (*Lepus tolai*) in China; however, the survey failed to detect *N. caninum* DNA from the tissues [4]. In spite of the worldwide distribution and broad host range of *N. caninum*, no data on the genetic characterization of rabbit-derived *N. caninum* isolates are available internationally.

The aim of this study was to determine the occurrence and genetic characterization of *T. gondii* and *N. caninum* from slaughtered domestic rabbits in Henan Province, Central China.

## Materials and methods

### Ethics statement

The research protocol was reviewed and approved by the Research Ethics Committee of Henan University of Science and Technology.

### Specimens

Between January 2017 and October 2018, brain and heart samples of 470 slaughtered domestic rabbits were collected from seven food markets in Luoyang, Zhengzhou, and Nanyang cities, Henan Province, central China. Each sample was placed into an individual clean self-sealing bag, and the information on the sex, breed, and market of animals was also recorded. All the tissues collected were frozen at −20 °C.

### Detection of *T. gondii* and *N. caninum* DNA

Approximately 1−2 g tissue was taken selectively from different anatomic regions of each tissue, and the specimen was subsequently homogenized in 3 mL of sterile phosphate-buffered saline (PBS). Genomic DNA was extracted from 300 μL of each homogenized tissue suspension using the commercial TIANamp Genomic DNA kit (TianGen, Beijing, China). Extracted DNA was stored at −20 °C until analysis. Molecular detections were carried out by nested PCR amplification based on the *T. gondii* B1 gene and *N. caninum* NC5 gene, respectively, as described previously [17, 18]. DNA of the *T. gondii* RH strain and *N. caninum* Nc-LY-Cow1 strain [19] was used in each analysis as the positive control, respectively. Distilled water was used in each analysis as the negative control.

### Genotype identification

*Toxoplasma gondii* genotyping was carried out using the PCR-RFLP method based on genetic markers SAG1, (3′ + 5′) SAG2, alt. SAG2, SAG3, BTUB, GRA6, c22-8, c29-2, L358, PK1, and Apico, as described previously [22]. The ITS1 region of the *N. caninum* NC5 gene positive DNA samples was amplified by nested PCR using the primers NN1 and NN2 as external primers, and NP1 and NP2 as internal primers, as described previously [10]. Multilocus microsatellite genotyping of *N. caninum* was done based on genetic markers MS4, MS5, MS6A, MS7, MS8, MS10, and MS12, as described previously [20]. DNA of *N. caninum* Nc-LY-Cow1 and distilled water were used in each analysis as the positive and negative controls, respectively. Two-directional sequencing of positive PCR products was done by Sangon Biotech Co., Ltd., (Shanghai, China). Nucleotide sequences obtained were aligned with each other and with available sequences in GenBank, using ClustalX 2.0, and the default setting, with manual adjustment. A neighbor-joining tree based on the ITS1 sequences was generated using MEGA7 software. The evolutionary distances were computed using the maximum composite likelihood method, and the reliability of branches in the tree was assessed by bootstrap analysis using 1000 replicates.

### Statistical analysis

Chi-square analysis was performed to assess the correlation between the occurrence of *T. gondii* and *N. caninum* DNA and the sex, breed, and region of rabbits using SPSS, version 11.5 (Statistical Package for the Social Sciences).

## Results and discussion

The occurrence rates of *T. gondii* and *N. caninum* DNA in slaughtered domestic rabbits in this study are presented in Table 1. DNA of *T. gondii* and *N. caninum* was detected in 13 (2.8%) and 10 (2.1%) of 470 rabbits, respectively. Co-infection with *T. gondii* and *N. caninum* was found in one rabbit (0.2%). The occurrence rate of *T. gondii* DNA in domestic rabbits in this study was lower than that (6.4%) in wild rabbits from Shandong Province, eastern China [4]. To our knowledge, this study is the first molecular evidence of *N. caninum* DNA in rabbits in China. The survey conducted by Cong et al. [4] failed to detect *N. caninum* DNA from wild rabbits and showed a seroprevalence rate of 0.8% in Shangdong Province, China. The occurrence rate (2.1%) of *N. caninum* DNA in domestic rabbits in this study was similar to that (2.8%) in cottontail rabbits (*Sylvilagus floridanus*) in Italy [26], but lower than that (10.5%) in wild rabbits (*Oryctolagus cuniculus*) in the UK [12].

**Table 1:** Occurrence of *Toxoplasma gondii* and *Neospora caninum* DNA and risk factors for infection in domestic rabbits in Henan Province, central China, based on PCR.

Variable	No. of rabbits	*T. gondii* DNA positive rabbits (%)	*p*-value	*N. caninum* DNA positive rabbits (%)	*p*-value
Region					
Zhengzhou	126	3 (2.4)	>0.05	2 (1.6)	>0.05
Luoyang	248	8 (3.2)		6 (2.4)	
Nanyang	96	2 (2.1)		2 (2.1)	
Breed					
Chinese rabbit	324	9 (2.8)	>0.05	8 (2.5)	>0.05
New Zealand rabbit	146	4 (2.7)		2 (1.4)	
Sex					
Male	251	6 (2.4)	>0.05	4 (1.6)	>0.05
Female	219	7 (3.2)		6 (2.7)	
Total	470	13 (2.8)		10 (2.1)	

Similar occurrence rates of *T. gondii* and *N. caninum* DNA were found in the different region, breed, or sex groups, with no significant differences (*p* > 0.05). In different regions, the occurrence rates were 2.1 ~ 3.2% for *T. gondii* and 1.6 ~ 2.4% for *N. caninum*, respectively. The occurrence rates in Chinese rabbits and New Zealand rabbits were 2.8% and 2.7% for *T. gondii*, 2.5% and 1.4% for *N. caninum*, and in male and female rabbits 2.4% and 3.2% for *T. gondii*, and 1.6% and 2.7% for *N. caninum*, respectively (Table 1).

In the present study, *T. gondii* DNA was detected mostly in brain tissue (10/13), consistent with a previous report by de Lima et al. [5]. *N. caninum* DNA was detected mostly in hearts (8/10), like the findings reported by Gondim et al. [11] in sparrows, and indicating that the heart should be included in molecular epidemiology studies of *N. caninum*.

In this study, only 1 out of 13 *T. gondii-*positive samples gave complete genotyping results at all 11 gene loci, and was identified to ToxoDB genotype #9, and two samples were genotyped at four and two genetic loci, respectively (Table 2). In China, ToxoDB genotype #9 is a predominant genotype, and has been found previously in a number of hosts in different regions including Henan Province [16, 23]. However, data on the genetic characterization of rabbit-derived *T. gondii* isolates in China are limited. So far, only two studies have reported one genotype III isolate from domestic rabbits [27] and two ToxoDB genotype #9 isolates from wild rabbits [4] in China. These data suggest that ToxoDB genotype #9 may be a prevalent lineage in rabbits in China. Further studies on a larger number of samples collected from different regions are needed to understand the genetic diversity of *T. gondii* from rabbits in China.

**Table 2:** Multilocus genotyping of *Toxoplasma gondii* from domestic rabbits in Henan Province, central China.

Isolate ID	Host	Location	SAG1	5′ + 3′ SAG2	alt. SAG2	SAG3	BTUB	GRA6	C22-8	C29-2	L358	PK1	Apico	Genotype
GT1, reference	Goat	USA	I	I	I	I	I	I	I	I	I	I	I	Type I, ToxoDB #10
PTG, reference	Sheep	USA	II/III	II	II	II	II	II	II	II	II	II	II	Type II, ToxoDB #1
CTG, reference	Cat	USA	II/III	III	III	III	III	III	III	III	III	III	III	Type III, ToxoDB #2
MAS, reference	Human	France	u-1^a^	I	II	III	III	III	u-1	I	I	III	I	ToxoDB #17
TgPHN1	Pig	Henan	u-1	II	II	III	III	II	II	III	II	II	I	ToxoDB #9 [19]
TgRbHN1	Rabbit	Luoyang	u-1	II	II	III	III	II	II	III	II	II	I	ToxoDB #9
TgRbHN2	Rabbit	Luoyang	u-1	II	nd	III	nd	II	nd	nd	nd	nd	nd	
TgRbHN3	Rabbit	Nanyang	u-1	nd	nd	III	nd	nd	nd	nd	nd	nd	nd	

a u-1 represents unique RFLP genotypes.

**Figure 1:**
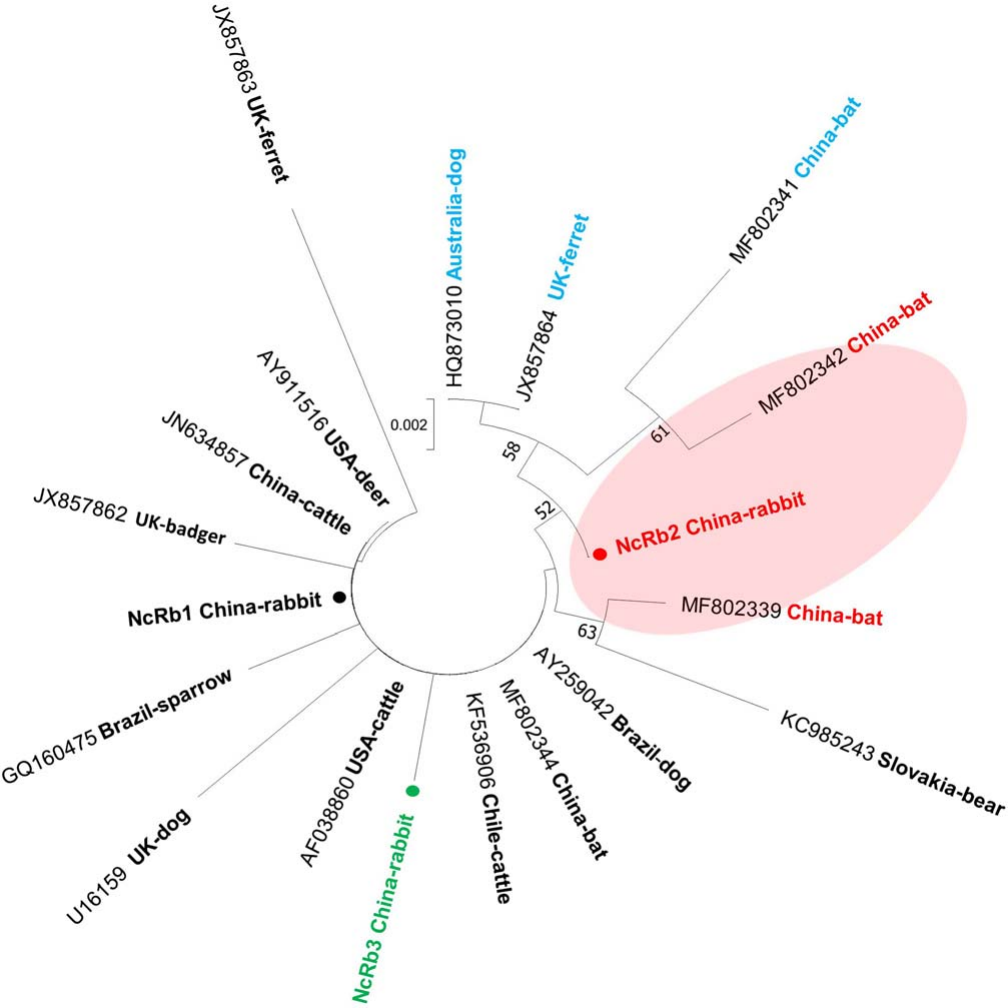
Phylogenetic relationships among NcRb1 − NcRb3 in this study and different representative sequences in GenBank, as inferred by a neighbor-joining analysis of the partial ITS1 region. Bootstrap values greater than 50% from 1000 pseudoreplicates are shown. The sequences in this study are marked by closed circles.

**Figure 2:**
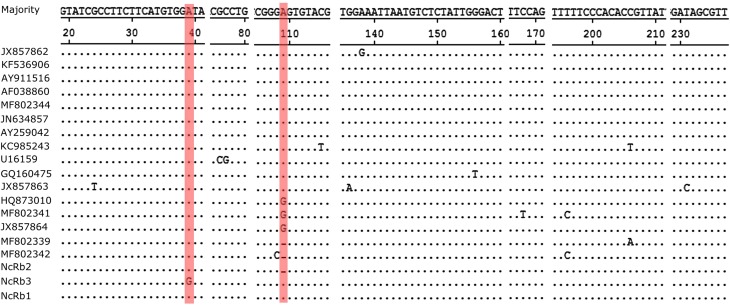
Sequence alignment of the partial ITS1 gene for the *Neospora caninum* isolates obtained in this study and reference sequences from GenBank. Dots (·) indicate identical nucleotides related to the sequence of JX857862 (first row) at that position and dashes (–) indicate deletions.

ITS1 sequences from the 8 N*. caninum*-positive rabbits in this study showed 95−100% similarity with *N. caninum* sequences available in GenBank. Three different ITS1 sequences were identified, namely NcRb1 (from five animals, accession number MK510934), NcRb2 (from two animals, accession number MK510935), and NcRb3 (from one animal, accession number MK510936). Among these ITS1 sequences obtained in this study, NcRb1, identified from 5 out of 8 N*. caninum* ITS1-positive animals and in two out of three cities, was apparently predominant. NcRb1 was identical to the majority of reference sequences available in GenBank. In the phylogenetic tree of the *N. caninum* ITS1 region, NcRb1 clusters with several representative sequences from domestic and wild animals (e.g., cattle, dogs, deer, and bats) worldwide, including two Chinese strains (JN634857 from cattle and MF802344 from bats) (Fig. 1). Minor sequence differences were observed in the ITS1 sequences NcRb2 and NcRb3, which contain a 1-bp deletion and a 1-bp mutation, respectively (Fig. 2). NcRb2 was located on an intermediate position between the two clusters where two bat-derived sequences from China also consisting of a 1-bp deletion (located as shown in Fig. 1).

Due to low DNA concentrations, the *N. caninum* NC5 gene positive DNA samples from six rabbits gave genotyping results only at the MS5 microsatellite locus. Two MS5 genotypes were identified, including the genotype (TA)_12_ (accession number MK510937) from four animals, and genotype (TA)_14_ (accession number MK510938) from two animals. After analyses on the complete dataset, the predominate genotype (TA)_12_ has been reported previously from dogs in Luoyang, China [18], as well as from dogs and cattle in the United States, Germany, and Spain [20]. The MS5 (TA)_14_ genotype was found in China for the first time, and had previously been reported from dogs and cattle in South Korea, Iran, Spain, Portugal, Mexico, Argentina, and Brazil [2, 3, 14, 20, 21]. In addition, MS5 genotypes (TA)_8_ and (TA)_9_ have been reported from cattle in the same region [17]. The above-mentioned analysis on ITS1 and MS5 sequences reveals genetic diversity of *N. caninum* in Henan Province, central China.

## Conclusions

In conclusion, this is the first molecular evidence of *N. caninum* DNA in rabbits in China. Genetic characterization of rabbit-derived *N. caninum* was determined based on the ITS1 region and MS5 locus, and the results reveal genetic diversity. One *T. gondii* isolate detected was identified as ToxoDB genotype #9 which is the major lineage in China. These findings provide new genetic information on both parasites especially *N. caninum* in rabbits, and have important implications for a better understanding of the genetic diversity of the parasites in China.

## References

[1] Alvarado-Esquivel C, Torres-Berumen JL, Estrada-Martinez S, Liesenfeld O, Mercado-Suarez MF. 2011 *Toxoplasma gondii* infection and liver disease: a case-control study in a northern Mexican population. Parasites & Vectors, 4, 75. 21569516 10.1186/1756-3305-4-75 PMC3105944

[2] Basso W, Schares S, Barwald A, Herrmann DC, Conraths FJ, Pantchev N, Vrhovec MG, Schares G. 2009 Molecular comparison of *Neospora caninum* oocyst isolates from naturally infected dogs with cell culture-derived tachyzoites of the same isolates using nested polymerase chain reaction to amplify microsatellite markers. Veterinary Parasitology, 160(1–2), 43–50. 19084341 10.1016/j.vetpar.2008.10.085

[3] Brom PR, Regidor-Cerrillo J, Collantes-Fernandez E, Ortega-Mora LM, Guimaraes MS, da Silva AC. 2014 Genetic characterisation of *Neospora caninum* strains from clinical samples of zebuine foetuses obtained in abattoirs in Goias, Brazil. Veterinary Parasitology, 204(3–4), 381–387. 24893690 10.1016/j.vetpar.2014.05.011

[4] Cong W, Zhou CX, Chen L, Zou Y, Wang WL, Meng QF, Qian AD. 2018 *Toxoplasma gondii* and *Neospora caninum* in Tolai Hares (*Lepus tolai*) intended for human consumption in China: seroprevalence, DNA detection, and genotyping. Foodborne Pathogens and Disease, 15(9), 544–547. 29782189 10.1089/fpd.2018.2436

[5] de Lima DC, Santos Ade S, da Silva LT, de Melo RP, da Silva JG, Junior JW, Mota RA. 2016 Occurrence of *Toxoplasma gondii* in domestic rabbits of Northeastern Brazil. Acta Parasitologica, 61(3), 500–507. 27447212 10.1515/ap-2016-0066

[6] do Nascimento LC, Pena HFJ, Leite Filho RV, Argenta FF, Alves BF, Oliveira S, Gennari SM, Driemeier D. 2017 Rare case of acute toxoplasmosis in a domestic rabbit (*Oryctolagus cuniculus*) in Brazil associated with the type Br III Brazilian clonal lineage of *Toxoplasma gondii*. Parasitology Research, 116(10), 2873–2876. 28849263 10.1007/s00436-017-5600-1

[7] Dubey JP, Carpenter JL, Speer CA, Topper MJ, Uggla A. 1988 Newly recognized fatal protozoan disease of dogs. Journal of the American Veterinary Medical Association, 192(9), 1269–1285. 3391851

[8] Dubey JP. 2010 Toxoplasmosis of animals and humans, 2nd edn CRC Press, Taylor & Francis Group: Boca Raton, Florida pp. 1–117, 313.

[9] Dubey JP, Hemphill A, Calero-Bernal R, Schares G. 2017 Neosporosis of animals. CRC Press: Boca Raton, Florida.

[10] Ellis JT, Amoyal G, Ryce C, Harper PA, Clough KA, Homan WL, Brindley PJ. 1998 Comparison of the large subunit ribosomal DNA of *Neospora* and *Toxoplasma* and development of a new genetic marker for their differentiation based on the D2 domain. Molecular and Cellular Probes, 12(1), 1–13. 9584073 10.1006/mcpr.1997.0143

[11] Gondim LS, Abe-Sandes K, Uzeda RS, Silva MS, Santos SL, Mota RA, Vilela SM, Gondim LF. 2010 *Toxoplasma gondii* and *Neospora caninum* in sparrows (*Passer domesticus*) in the Northeast of Brazil. Veterinary Parasitology, 168(1–2), 121–124. 19879051 10.1016/j.vetpar.2009.09.055

[12] Hughes JM, Thomasson D, Craig PS, Georgin S, Pickles A, Hide G. 2008 *Neospora caninum*: detection in wild rabbits and investigation of co-infection with *Toxoplasma gondii* by PCR analysis. Experimental Parasitology, 120(3), 255–260. 18703054 10.1016/j.exppara.2008.07.011

[13] Luo H, Li K, Shahzad M, Zhang H, Lan Y, Xiong X. 2017 Seroprevalence of *Toxoplasma gondii* infection in wild boars, wild rabbits, and wild chickens in Hubei Province, China. Korean Journal of Parasitology, 55(1), 85–88. 28285512 10.3347/kjp.2017.55.1.85 PMC5365258

[14] Medina-Esparza L, Regidor-Cerrillo J, Garcia-Ramos D, Alvarez-Garcia G, Benavides J, Ortega-Mora LM, Cruz-Vazquez C. 2016 Genetic characterization of *Neospora caninum* from aborted bovine foetuses in Aguascalientes, Mexico. Veterinary Parasitology, 228, 183–187. 27692324 10.1016/j.vetpar.2016.09.009

[15] Meng QF, Wang WL, Ni XT, Li HB, Yao GZ, Sun XL, Wang WL, Cong W. 2015 Seroprevalence of *Encephalitozoon cuniculi* and *Toxoplasma gondii* in domestic rabbits (*Oryctolagus cuniculus*) in China. Korean Journal of Parasitology, 53(6), 759–763. 26797446 10.3347/kjp.2015.53.6.759 PMC4725227

[16] Qian WF, Yan WC, Wang TQ, Shao XD, Zhai K, Han LF, Lv CC. 2015 Genetic characterization of *Toxoplasma gondii* from domestic animals in Central China. Tropical Biomedicine, 32(3), 540–544. 26695215

[17] Qian WF, Yan WC, Wang TQ, Zhai K, Han LF, Lv CC. 2015 Prevalence and genetic characterization of *Toxoplasma gondii* in pet dogs in Central China. Korean Journal of Parasitology, 53(1), 125–128. 25748720 10.3347/kjp.2015.53.1.125 PMC4384793

[18] Qian W, Wang T, Yan W, Han L, Zhai K, Duan B, Lv C. 2016 Occurrence and first multilocus microsatellite genotyping of *Neospora caninum* from naturally infected dogs in dairy farms in Henan, Central China. Parasitology Research, 115(8), 3267–3273. 27230015 10.1007/s00436-016-5142-y

[19] Qian W, Wang T, Yan W, Zhang M, Han L, Xue R, Song S, Lv C. 2017 Seroprevalence and first multilocus microsatellite genotyping of *Neospora caninum* in dairy cattle in Henan, Central China. Veterinary Parasitology, 244, 81–84. 28917323 10.1016/j.vetpar.2017.07.022

[20] Regidor-Cerrillo J, Diez-Fuertes F, Garcia-Culebras A, Moore DP, Gonzalez-Warleta M, Cuevas C, Schares G, Katzer F, Pedraza-Diaz S, Mezo M, Ortega-Mora LM. 2013 Genetic diversity and geographic population structure of bovine *Neospora caninum* determined by microsatellite genotyping analysis. PLoS One, 8(8), e72678. 23940816 10.1371/journal.pone.0072678 PMC3735528

[21] Salehi N, Gottstein B, Haddadzadeh HR. 2015 Genetic diversity of bovine *Neospora caninum* determined by microsatellite markers. Parasitology International, 64(5), 357–361. 25988829 10.1016/j.parint.2015.05.005

[22] Su C, Shwab EK, Zhou P, Zhu XQ, Dubey JP. 2010 Moving towards an integrated approach to molecular detection and identification of *Toxoplasma gondii*. Parasitology, 137(1), 1–11. 19765337 10.1017/S0031182009991065

[23] Wang H, Zhang L, Ren Q, Yu F, Yang Y. 2017 Diagnosis of Swine Toxoplasmosis by PCR and Genotyping of *Toxoplasma gondii* from pigs in Henan, Central China. BMC Veterinary Research, 13(1), 152. 28569215 10.1186/s12917-017-1079-3 PMC5452427

[24] Wang S, Yao Z, Li L, Pan Y, Li P, Nan X, Xie Q, Zhang Z. 2018 Seroprevalence of *Toxoplasma gondii* and *Encephalitozoon cuniculi* among domestic rabbits in Central China. Parasite, 25, 9. 29521259 10.1051/parasite/2018010 PMC5844235

[25] Wang T, Han Y, Pan Z, Wang H, Yuan M, Lin H. 2018 Seroprevalence of *Toxoplasma gondii* infection in blood donors in mainland China: a systematic review and meta-analysis. Parasite, 25, 36. 30040610 10.1051/parasite/2018037 PMC6057739

[26] Zanet S, Palese V, Trisciuoglio A, Canton Alonso C, Ferroglio E. 2013 *Encephalitozoon cuniculi*, *Toxoplasma gondii* and *Neospora caninum* infection in invasive eastern cottontail rabbits *Sylvilagus floridanus* in Northwestern Italy. Veterinary Parasitology, 197(3–4), 682–684. 23747104 10.1016/j.vetpar.2013.05.014

[27] Zhou Y, Zhang H, Cao J, Gong H, Zhou J. 2013 Isolation and genotyping of *Toxoplasma gondii* from domestic rabbits in China to reveal the prevalence of type III strains. Veterinary Parasitology, 193(1–3), 270–276. 23261088 10.1016/j.vetpar.2012.11.031

